# Student approaches for learning in medicine: What does it tell us about the informal curriculum?

**DOI:** 10.1186/1472-6920-11-87

**Published:** 2011-10-21

**Authors:** Jianzhen Zhang, Raymond F Peterson, Ieva Z Ozolins

**Affiliations:** 1 Centre for Medical Education Research and Scholarship, School of Medicine, The University of Queensland, 288 Herston Road, Herston QLD 4006 Australia; 2 Faculty of Health Sciences. The University of Adelaide, 5005 Australia; 3 Faculty of Health, Queensland University of Technology, Victoria Park Road, Kelvin Grove, QLD 4059 Australia

## Abstract

**Background:**

It has long been acknowledged that medical students frequently focus their learning on that which will enable them to pass examinations, and that they use a range of study approaches and resources in preparing for their examinations. A recent qualitative study identified that in addition to the formal curriculum, students are using a range of resources and study strategies which could be attributed to the informal curriculum. What is not clearly established is the extent to which these informal learning resources and strategies are utilized by medical students. The aim of this study was to establish the extent to which students in a graduate-entry medical program use various learning approaches to assist their learning and preparation for examinations, apart from those resources offered as part of the formal curriculum.

**Methods:**

A validated survey instrument was administered to 522 medical students. Factor analysis and internal consistence, descriptive analysis and comparisons with demographic variables were completed. The factor analysis identified eight scales with acceptable levels of internal consistency with an alpha coefficient between 0.72 and 0.96.

**Results:**

Nearly 80% of the students reported that they were overwhelmed by the amount of work that was perceived necessary to complete the formal curriculum, with 74.3% believing that the informal learning approaches helped them pass the examinations. 61.3% believed that they prepared them to be good doctors. A variety of informal learning activities utilized by students included using past student notes (85.8%) and PBL tutor guides (62.7%), and being part of self-organised study groups (62.6%), and peer-led tutorials (60.2%). Almost all students accessed the formal school resources for at least 10% of their study time. Students in the first year of the program were more likely to rely on the formal curriculum resources compared to those of Year 2 (*p *= 0.008).

**Conclusions:**

Curriculum planners should examine the level of use of informal learning activities in their schools, and investigate whether this is to enhance student progress, a result of perceived weakness in the delivery and effectiveness of formal resources, or to overcome anxiety about the volume of work expected by medical programs.

## Background

It has long been acknowledged that medical students frequently focus their learning on that which will enable them to pass examinations [1], and that they use a range of study approaches and resources in preparing for their examinations [2]. Modern problem-based learning (PBL) curricula were designed so that students were provided with a more challenging learning environment which increased motivation to learn, and enabled students to develop self-directed learning approaches which were deemed to be part of the lifelong learning skills they would need as part of the medical profession [3]. Typically programs are designed to enable students to build on their existing knowledge base, and apply the new knowledge from relevant disciplines in the analysis of a clinical scenario. The learning environment and the sequencing of the educational sessions, including tutorials, lectures and practicals, is often guided by the plan to assist students in the integration of the disciplines and to develop a more meaningful understanding of the material under discussion. Hence, it would be expected that students' approaches to studying would be influenced by these opportunities provided through these learning environments [4] and that this would lead to a deep learning approach.

Some problem-based learning (PBL) medical curricula have a formal curriculum and resources which are delivered to all students as part of the educational program. In a recent qualitative study [2], it was identified that in addition to the formal curriculum, students are using a range of resources and study strategies, such as past exam papers, past student notes, peer-led tutorials, self-organized study groups, discussions with students from previous cohorts and accessing PBL tutor guides, which could be attributed to the informal curriculum. Preparation for examinations was identified by the students in these focus groups as being a major part of their work. What is not clearly established in these focus groups is the extent to which these informal learning resources and strategies are utilized by medical students. In addition, there is concern that the emphasis by students on preparation for examinations results in more superficial learning and retention of knowledge to pass these examinations; which is in conflict with the learning processes developing during the semesters.

The concepts of the formal, informal and hidden curricula are not new. Hafferty [5] defined the formal curriculum as 'the stated, intended, and formally offered and endorsed curriculum'; the informal curriculum as 'an unscripted, predominantly *ad hoc*, and highly interpersonal form of teaching and learning that takes place among and between faculty and students', and the hidden curriculum as 'a set of influences that function at the level of organisational structure and culture'. In medical education, research on the informal curriculum has explored a range of topics, including continuing medical education, the role of patient centered and doctor centered care, cultural competency, multicultural education, teaching in medical education, and the student - teacher relationship [5-8]. The informal curriculum, particularly in the context of a professional medical training program, has also been explored in the context of student experiences of ethical and cultural issues and professionalism through their education and training in a range of clinical settings with experienced clinicians [7-11].

Whilst significant understanding of how students learn has improved teaching, learning and assessment approaches in medical programs, these activities relate more directly to the design and delivery of the formal curriculum. There is very little reported on the concepts of the informal and hidden curriculum from the student perspective, particularly in relation to the approaches used by students in their learning and preparation for written examinations in medical school [2]. Students in one graduate-entry medical program were aware that a formal and informal curriculum did exist to a greater degree in medicine than in their undergraduate degrees [2]. Furthermore, a range of approaches and factors, some of which were well outside the scope of the intended or taught curriculum, may influence the way in which students approach their learning of the formal curriculum. These included past exam papers, peer-led tutorials, self-organized study groups, discussions with students from previous cohorts, past student notes that are formally distributed amongst the student population but not to staff, and informal discussions with clinical teachers and PBL tutors that go beyond the formal curriculum. Student-student interactions contribute significantly to the informal curriculum.

The purpose of this study was to develop a survey instrument to (1) examine its construct validity and reliability; (2) establish the extent to which students use various learning or study methods that could be attributed to the formal and informal curriculum in their learning and preparation for examinations and (3) explore relationships between specific student characteristics and the use of formal and informal curricula. The formal curriculum was defined as all educational activities which were coordinated and formally delivered within the medical program. The informal curriculum included all activities which were initiated by the students to address their learning needs in the program, including assisting them to pass the examinations. The rationale for this study was to provide evidence of and to inform the curriculum designers and course planners about the extent to which students use the formal and informal curriculum, to recognize the nature of this learning enterprise undertaken by students as part of their self-directed learning, and to develop an understanding of the competing demands of the formal and informal curriculum for students. Students individually and in groups developing strategies to pass examinations are not new, but the fact that preparing for examinations was identified by students to be a significant part of their learning, led to the need to establish the extent to which this is undertaken by a large student cohort, and the scope of activities completed in this process.

## Methods

### Participants and study setting

A cross-sectional quantitative study was conducted among students in PBL groups from years 1 and 2 in a 4-year graduate-entry medical program at the School of Medicine at the University of Queensland (UQ) in 2008. The formal curriculum at UQ includes 5 hours of PBL per week in two sessions, supported by weekly small group clinical skills tutorials, anatomy and/or pathology tutorials in most weeks, and up to eight hours of lectures or clinical symposia per week. Table 1 presents the characteristics of the participants. Of 522 participants (249 males and 247 females), 323 were from year 1 and 199 from year 2. The response rate was 88.5% for year 1 and 62.2% for year 2. Participants had a mean age of 24.5 years (range 18-54 years). The majority of students were of Australian origin (64%), and had a biological science (58%) or health professional background (20%). The sample was representative as the distribution was similar to the data for 2001-2003 cohorts of the program [12]. There were some missing data from the total sample including age (3.1%), gender (3.1%), country of origin (2.7%) and previous degree (10.0%).

**Table 1:** Participant characteristics of year 1 and year 2 medical students

	Year 1 N (%)	Year 2 N (%)	Total
**Number**	323 (61.9)	199 (38.1)	522 (100.0)
**Mean Age**	24.2(18-54)	24.9(21-47)	24.5(18-54)
Young (18-24)	224 (69.3)	116 (58.3)	340 (65.1)
Mid (25-29)	67 (20.7)	57 (28.6)	124 (23.8)
Old (> 30)	24 (7.4)	18 (9.1)	42 (8.0)
Missing	8 (2.5)	8 (4.0)	16 (3.1)
**Gender**			
Male	165 (51.1)	94 (47.2)	259 (49.6)
Female	149 (46.1)	98 (49.2)	247 (47.3)
Missing	9 (2.8)	7 (3.5)	16 (3.1)
			
**Country of Origin**			
Australia	207 (64.1)	129 (64.8)	336 (64.4)
Overseas	109 (33.7)	63 (31.7)	172 (32.9)
Missing	7 (2.2)	7 (3.5)	14 (2.7)
**Previous degree**			
Biological science	194 (60.1)	109 (54.8)	303 (58.0)
Health professions	66 (20.4)	36 (18.1)	102 (19.5)
Physical science	12 (5.2)	6 (3.0)	18 (3.4)
Non-science	32 (9.9)	15 (7.5)	47 (9.0)
Missing	19 (5.9)	33 (16.6)	52 (10.0)

### Development of the questionnaire

The Formal and Informal Curriculum Questionnaire (FICQ) was developed using the data obtained from the focus group discussions (FGDs) reported in a previous study [2]. The questionnaire comprised two sections. The first section included 40 items covering 11 domains addressing student use of and perceptions about the value of the formal and informal curriculum, the contribution of a range of learning activities to their study, and the proportion of time devoted to the learning activities. The second section consisted of 10 items and collected student background demographic information including age, gender, country of origin, year of study in the MBBS program, and first degree major. The 11 domains of the questionnaire were:

(1) Importance of the formal and informal curriculum (6 items);

(2) Proportion of time devoted to the learning activities (6 items);

(3) Perceptions of the formal and informal curriculum for the medical program (5 items);

(4) Perceptions of the formal curriculum (2 items);

(5) Reasons for using the informal curriculum (2 items);

(6) Value of using peer-led tutorial groups (4 items);

(7) Value of using self-organized study groups (4 items);

(8) Value of using student notes from past students (4 items);

(9) Value of accessing past exam papers (2 items);

(10) Value of talking to students from previous cohorts (4 items); and

(11) Value of accessing PBL tutor guides (1 item).

Items in the domain 'Importance of the formal and informal curriculum' used a six-point response format with the responses 'very important,' 'fairly important', 'not very important', 'not at all important', 'I don't think about it', and 'not applicable' and for the purpose of data entry and data analysis were coded from 5 to 1 and 0 respectively. Items exploring the proportion of time devoted to different learning activities used a 10-100 percent scale measurement. Items related to Domains (3)-(11) used a six-point response format ranging from 'strongly agree' to 'strongly disagree' with a choice of "not applicable", and were coded from 6 to 1 and 0 respectively. The details of the items for domains (3)-(11) are described in Table 2.

**Table 2:** Results of factor loading from final Principal Component Analysis

	Components							
**Scales and Items**	**1**	**2**	**3**	**4**	**5**	**6**	**7**	**8**

**Perception of the formal and informal curriculum of medical program**								
1. I feel overwhelmed learning both the formal curriculum and the informal curriculum	.025	-.036	-.025	.120	**.814**	.084	.098	.047
2. I feel overwhelmed by the formal curriculum I am required to cover	-.029	-.045	-.065	.128	**.791**	.179	.046	.092
3. I do not think I would pass the exam without the informal curriculum	.045	.088	.203	.143	**.699**	-.118	.101	.071
4. I do not think I would be a good doctor without the informal curriculum	.058	.166	.196	.047	**.625**	-.195	-.018	.049
5. I trust other sources of information more than the learning objectives of the formal curriculum when deciding where to focus my learning	-.040	.047	.154	-.103	.483	-.317	-.074	.206
**Perception of the formal curriculum**								
1. The formal curriculum is clearly stated	.027	-.005	.044	-.025	-.073	**.874**	.025	.012
2. Most of what I need to know for the programme is covered on the formal curriculum	.059	-.053	.007	-.060	-.012	**.858**	-.058	.055
**Reasons for using the informal curriculum**								
1. To judge the right level of the learning that I need to do	.091	.041	.156	.034	.046	-.017	**.888**	-.026
2. To work out what is important to pass the exams	.122	.018	.170	.049	.115	-.006	**.873**	.088
**Value of using peer-led tutorial groups**								
1. Reassure me that I am on the right track	**.947**	.056	.076	.140	.007	.033	.106	.028
2. Tell me what is relevant to learn to pass exam	**.940**	.039	.066	.117	.003	.013	.083	.044
3. Encourage a collegiate learning environment	**.910**	.090	.071	.134	-.004	.025	.036	.012
4. Focus my learning on what is important to be a good doctor	**.903**	.091	.134	.111	.078	.036	.030	-.049
								
**Value of using self-organised study groups**								
1. Reassure me that I am on the right track	.044	**.956**	.040	.111	.025	-.032	.029	.038
2. Tell me what is relevant to learn to pass exams	.075	**.929**	.070	.088	.023	-.015	.057	.078
3. Encourage a collegiate learning environment	.072	**.922**	.061	.129	.026	-.034	.009	.040
4. Focus my learning on what is important to be a good doctor	.063	**.901**	.108	.068	.096	-.008	-.016	-.025
**Value of using student notes from past students**								
1. Save time in learning	.055	.046	**.903**	.097	.044	.010	.081	.073
2. Make sure what to study	.055	.083	**.898**	.120	.108	.033	.117	.072
3. Focus on what is important to know for exams	.064	.064	**.883**	.131	.082	.000	.171	.067
4. Focus on what is important to be a good doctor	.160	.072	**.804**	-.008	.108	-.010	.025	-.035
								
**Value of accessing past exam papers**								
1. The only way I know what to learn	.023	.017	.066	.083	.161	-.065	-.051	**.880**
2. A way I can work out what is important in the formal curriculum	.002	.092	.060	.141	.146	.131	.122	**.809**
**Value of talking to students from previous cohorts**								
1. Is the way I can work out what is important in the formal curriculum	.137	.065	.077	**.876**	.056	.004	.025	.126
2. Helps me work out how I am going with the program	.161	.155	.047	**.868**	.089	.033	.071	.003
3. Provides reassuring advice and encouragement	.175	.137	.070	**.862**	.102	.014	.078	-.059
4. Is the only way I know what to learn	.116	-.006	.092	**.739**	.134	-.133	-.052	.267
								
**Value of accessing PBL tutor guides**								
1. Give me more direction in my studies	-.116	.090	.115	.160	.027	-.024	-.010	-.016
**Total Variance Explained (%)**	**12.91**	**12.82**	**11.82**	**11.00**	**9.14**	**6.21**	**6.03**	**5.79**

An initial draft questionnaire was reviewed by the project team and piloted with two PBL groups (20 students) each selected at random from year 1 and year 2 respectively. Each group was provided with a checklist for feedback which was used to refine the questionnaire. Much of the feedback related to minor word changes to clarify statements. The final FICQ consisted of a 6-page A4 booklet. The questionnaires were distributed to each PBL group in Years 1 and 2 and then collected by PBL tutors.

### Data analysis

Descriptive statistics were performed for participants' characteristics and domains 1 and 2. Factor analysis using principal component analysis (PCA) with Varimax rotation and internal consistency were conducted to examine the construct validity and reliability for the domain scales (3)-(11). Domains 1-2 used different scales and were not included in this factor analysis. T-Test and ANOVA were used for statistical comparisons on the demographic data in section 2. SPSS (17.0) [13] was used for data analysis and the statistically significant difference of the means was identified at the 0.05 level.

### Ethics

Ethical clearance was received from the Behavioural and Social Sciences Ethical Review Committee, the University of Queensland. No funding was sought for the study.

## Results

### Construct validity and reliability of the instrument

From the initial analysis, the KMO value was 0.794 and Bartlett's Test was significant *(p *< .001). The final PCA produced 8 factor components with eigenvalues greater than 1, explaining a total of 75.7% of the variance, with individual factors contributing from 5.8% to 12.9%. Table 2 presents the final component loadings for all of the complete scale items and the retained factor loadings are highlighted in bold. From the PCA results, two items (one item from the scale of *Perception of the formal and informal curriculum *and one item from *Value of accessing PBL tutor guides*) were excluded because of low loadings (less than 0.5).

Table 3 presents mean scores, standard deviations and Cronbach's *α *reliability coefficients for the factorially derived scales. The PCA identified eight scales with acceptable levels of internal consistency: (1) Perceptions of the formal and informal curriculum for the medical program (5 items), *α *= 0.75; (2) Perceptions of the formal curriculum (2 items), *α *= 0.77, (3) Reasons for using the informal curriculum (2 items), *α *= 0.81, (4) Value of using peer-led tutorial groups (4 items), *α *= 0.96, (5)Value of using self-organized study groups (4 items), *α *= 0.96, (6)Value of using student notes from past students (4 items); *α *= 0.92, (7) Value of accessing past exam papers (2 items), *α *= 0.72, and (8) Value of talking to students from previous cohorts (4 items), *α *= 0.89.

**Table 3:** Mean scores, standard deviations and Cronbach's Alpha reliability coefficients for the factorially derived scales

Scales	Number of Cases	Number of Items (score range)	Mean (SD)	Cronbach's Α
1. Perception of the formal and informal curriculum of medical program	481	4 (4-24)	17.71 (3.77)	0.75
2. Perception of the formal curriculum	493	2 (2-12)	6.23 (2.24)	0.77
3. Reasons for using the informal curriculum	500	2 (2-12)	9.96 (1.76)	0.81
4. Value of using peer-led tutorial groups	372	4 (4-24)	19.29 (3.04)	0.96
5. Value of using self-organised study groups	386	4 (4-24)	18.26 (3.16)	0.96
6. Value of using student notes from past students	460	4 (4-24)	18.75 (3.62)	0.92
7. Value of accessing past exam papers	494	2 (2-12)	8.17 (2.12)	0.72
8. Value of talking to students from previous cohorts	464	4 (4-24)	16.94 (3.08)	0.89

"Not applicable" is excluded from each scale.

### Students' responses

Table 4 presents the proportion of the participants' responses and information on the individual items of each scale. Of the total students, 41.7% of the students (N = 504, Mean = 3.14) agreed (including moderately agree, strongly agree, and agree) that the formal curriculum was clearly stated, and 40.5% of the students (498, 3.08) believed that what they needed to know to complete the program was covered in the formal curriculum. There were 79.8% of the students (505, 4.56) were overwhelmed by the amount of work that was necessary to complete the formal curriculum, and 74.3% indicated that the informal curriculum helped them to pass the exams. The informal curriculum was perceived by 61.3% of students (493, 4.03) to be important in enabling them to become a good doctor, but 77.8% of the students (501, 4.51) believed that to learn both the formal and informal curriculum to pass the examinations increased their workload. Most students identified that the reasons for using the informal curriculum were to be able to work out what is important to pass the exams (93.2%) and to judge the right level of the learning that they need to do to pass examinations (94.3%). As also can be seen from Table 4, the *value *of the various approaches to help students learns is demonstrated in the high responses for most of the items in each of these domains. For example, peer led tutorials helped students know what was required for exams (97.1%), reassured students they were on the right track with their learning (96.3%), encouraged a collegiate learning environment (93.4%) and to a lesser extent the learning to be a good doctor (78.1%). This pattern of high positive responses was evident in each of these value domains.

**Table 4:** Proportion of the participants' responses and information on the individual item of eight scales

Scales and Items	N	Agree* (1+2+3)	Mean	Std. Deviation	Median	Range	95% Confidence Interval	
							
							Lower	Upper
**Perception of the formal and informal curriculum of medical program**								
1. I feel overwhelmed learning both the formal curriculum and the informal curriculum	501	77.8	4.51	1.18	5.00	5	4.41	4.61
2. I feel overwhelmed by the formal curriculum I am required to cover	505	79.8	4.56	1.16	5.00	5	4.46	4.66
3. I do not think I would pass the exam without the informal curriculum	508	74.3	4.56	1.32	5.00	5	4.45	4.68
4. I do not think I would be a good doctor without the informal curriculum	493	61.3	4.03	1.37	4.00	5	3.91	4.15
								
**Perception of the formal curriculum**								
1. The formal curriculum is clearly stated	504	41.7	3.14	1.29	3.00	5	3.03	3.26
2. Most of what I need to know for the programme is covered on the formal curriculum	498	40.5	3.08	1.21	3.00	5	2.98	3.19
								
**Reasons for using the informal curriculum**								
1. To judge the right level of the learning that I need to do	503	94.3	5.02	1.01	5.00	5	4.93	5.11
2. To work out what is important to pass the exams	500	93.2	4.95	1.00	5.00	5	4.86	5.03
**Value of using peer-led tutorial groups**								
1. Reassure me that I am on the right track	400	96.3	5.00	0.91	5.00	5	4.91	5.09
2. Tell me what is relevant to learn to pass exam	402	97.1	5.21	0.88	5.00	5	5.13	5.30
3. Encourage a collegiate learning environment	391	93.4	4.76	0.10	5.00	5	4.66	4.86
4. Focus my learning on what is important to be a good doctor	388	78.1	4.27	1.14	4.00	5	4.16	4.39
**Value of using self-organised study groups**								
1. Reassure me that I am on the right track	407	93.3	4.74	0.94	5.00	5	4.65	4.83
2. Tell me what is relevant to learn to pass exams	405	85.4	4.52	1.07	5.00	5	4.41	4.62
3. Encourage a collegiate learning environment	404	96.3	4.94	0.95	5.00	5	4.85	5.03
4. Focus my learning on what is important to be a good doctor	402	72.1	4.07	1.17	4.00	5	3.96	4.19
								
**Value of using student notes from past students**								
1. Save time in learning	476	91.2	5.01	1.15	5.00	5	4.90	5.11
2. Make sure what to study	475	94.6	5.02	1.01	5.00	5	4.93	5.11
3. Focus on what is important to know for exams	476	91.0	4.91	1.06	5.00	5	4.82	5.01
4. Focus on what is important to be a good doctor	468	56.2	3.81	1.23	4.00	5	3.70	3.93
								
**Value of accessing past exam papers**								
1. The only way I know what to learn	504	42.0	3.48	1.30	3.00	5	3.36	3.59
2. A way I can work out what is important in the formal curriculum	498	89.9	4.69	1.12	5.00	5	4.59	4.79
**Value of talking to students from previous cohorts**								
1. Is the way I can work out what is important in the formal curriculum	481	81.1	4.24	1.01	4.00	5	4.15	4.33
2. Helps me work out how I am going with the program	474	86.3	4.41	0.96	4.00	5	4.33	4.50
3. Provides reassuring advice and encouragement	481	96.5	4.78	0.91	5.00	5	4.70	4.86
4. Is the only way I know what to learn	479	41.9	3.47	1.15	3.00	5	3.36	3.57

*1 = strongly agree, 2 = moderately agree, 3 = agree

When we examined the proportions of study time that students devoted to formal and informal study activities, we found the activities used by most students were the school resources (94.4%) and past exam papers (92.1%). As may be expected, of the 94.4% students who accessed the school resources: approximately one in four students devoted only 10% of their study time to these resources, and one in five students devoted around 20% of their time. Only 12.5% of the students used the school resources for more than 60% of their study time. Past exam papers, which also contribute to the formal curriculum, were accessed by 92.1% of students, with almost half of the students devoting 10% of their study time to this resource. Most students also used a range of other informal activities for learning including past student notes (85.8%), PBL tutor guides (62.7%), self-organised study groups (62.6%) and peer-led tutorials (60.2%). The proportion of time students spent on each of these activities varied. For example, of the 62.7% of students who used PBL study guides, 48.2% used them for 10% of their time, and 11.1% for 20% of their time. The proportion of time spent on past student notes was spread over a wider range with 9.5% of students spending 50% of their time on this activity.

### Associations between student characteristics and perceptions

Some statistical differences in student perceptions of the value of the formal and informal curriculum were evident based on gender, age group, year of study and country of origin. Female students were more likely to perceive that the formal and the informal curriculum of the medical program were important for their learning (Scale 1, *p *= 0.001). Students in the 18-24 age group were more likely than the mid age group (25-29) to have a positive perception of the formal curriculum (Scale 2, *p *= 0.026), and valued using past student notes for their learning and exam preparation highly (Scale 6, *p *= 0.035).

A comparison of student perceptions between Year 1 and Year 2 indicated that the students in Year 1 were more likely to consider the formal curriculum to be clearly stated and to cover most of what they need to know for the program (Scale 2, *p *= 0.008), and valued accessing past exam papers as a way to learn (Scale 7, *p *= 0.001), whereas students from Year 2 valued self-organized study groups more highly (Scale 5, *p *= 0.015).

Students from overseas, when compared to the Australian students, were more likely to agree that accessing past exam papers was a way to know what to learn and work out what is important in the formal curriculum (Scale 7, *p *= 0.015). There was no statistically significant effect of prior degree on students' perceptions or use of the formal and informal curriculum (F = 0.166-1.493; *p *> 0.05).

## Discussion

This study used a questionnaire to help understand student perceptions of the formal and informal curriculum, and also identified some potential factors based on the initial qualitative study outcomes [2]. The eight scales were formally tested for their validity and reliability. Only two items were eliminated from this analysis. Relatively little research attention has been given to the development of standardised and psychometrically sound scales for measuring student's perceptions of the formal and informal curriculum in medical programs. The eight reliable scales are suitable for further development and more widespread use in research aimed at understanding students' perceptions of formal and informal curriculum. Based on these outcomes, the instrument could be applied to similar contexts to extend understanding of the formal and informal curriculum with a particular focus on student learning and preparation for examinations.

A study reporting interviews with 14 medical students in an undergraduate medical program reported that these students feel anxious about the quantity of information required for medicine, and were unsure about the appropriate depth of knowledge and amounts of individual study required [14]. The study also recognised concern amongst students that the perceived work load constituted a barrier to high quality learning. The majority of students responding to our questionnaire also reported feeling overwhelmed by the amount of work necessary to complete the formal curriculum, and believed that the informal curriculum helped them to pass the examinations. In fact, over 90% of the students who used past student notes reported doing so to make sure that they knew what they should study, and because the notes 'saved time in learning' and 'focused on what is important for exams'. This raises the question as to whether the students revert to more strategic and possibly more surface approaches to accommodate the knowledge that is necessary in the program through this process. In addition, if students are overwhelmed throughout the semester this may lead to students learning in ways not intended by the curriculum planners. Of particular concern is the cumulative effort for students who felt that they needed to address both the formal and informal curriculum, as this increased their workload and clearly contributed to their sense of feeling overwhelmed. This conflict in learning expectations from the individual and the program is an area that needs further investigation.

Over half of the students reported that the formal curriculum was not clear and did not cover all of what they needed to know for the medical program. This perception needs to be examined more thoroughly. We have previously reported that students believed that the informal curriculum is important in learning how to be and think like doctors [2]. If this somewhat intangible aspect of medical education is not delineated in the formal curriculum, and is difficult to assess, then the perceptions of the students about the formal curriculum may not be a severe indictment. Alternatively, some concern may well arise for students engaged for the first time in a self-directed learning environment, where triggers from their PBLs are the only guides for what should be addressed, and minimal time is provided to direct teaching activities, for example lectures and tutorials.

Almost all medical students used the school resources and past exam papers to some extent in their learning activities, and in their preparation for the examinations. However, over 85% of students responding to the questionnaire reported accessing past student notes for some of their study, with more than a third using this informal resource as their predominant learning activity. Most students also used self-organized tutorial groups, PBL tutor guides, as well as peer-led tutorial groups as part of their learning. Formal distribution of tutor guides to students is not supported by the school; however on occasion individual tutors share the content of the guides with students with likely resultant flow-on effects through study groups. Although use of PBL tutor guides and past student notes could be considered bypassing the principles of self-directed learning, the benefits to students of interacting in study groups and peer-led tutorials should be emphasized. Bradshaw and Hendry's studies [15,16] confirmed that participation in a study group was supportive and helpful for students to identify learning needs, clarify misunderstandings, share explanations and summaries, motivate individual study, and consolidate their learning. We have reported previously that many students considered that the informal curriculum provides "richness" and depth in their learning and contributes to the acquisition of the skills needed for lifelong professional learning; these attributes are more likely to have come from the interactive components of the informal curriculum, be they with clinical teachers or with other students.

The students in the first year of the program were more likely to rely on the formal curriculum resources. Although a proportion of the students will have been familiar with each other through common undergraduate degrees, most first year medical students are likely to be entering a new environment, and would require a period of familiarization with the course and their own cohort before establishing the contacts to explore the resources of the informal curriculum. Students in the second year of the program are more likely to have established relationships through the PBL group structure and larger groups for practical sessions, and with increasing exposure to clinical experiences more likely to access the resources of the informal curriculum, the benefits of which increase over time and with experience. The difference in student opinion may simply reflect different stages of their learning. Since the informal curriculum is important for medical students learning, we are in a position to urge them to share these experiences, which is critical to their professional development [17].

The fact that students considered the formal curriculum overwhelming could indicate that they are experiencing overload in their learning, and are having to revert to more surface and strategic approaches to cope. Further research is needed to explore the answers to these questions.

The limitations of this study were that it addressed the outcomes of one medical school, and some of the learning activities derived from the initial qualitative study may not be representative of those in other medical schools. In addition, the response rate for year 2 students was lower than anticipated, and in the analysis there were some missing data for the student characteristics variables. Future studies in other medical schools could explore the extent to which the informal curriculum is similar or different to the data and outcomes identified in this study, so that a better understanding is developed of the informal curriculum on student learning and preparation for examinations.

## Conclusions

If one of the goals of medical education was to encourage students to be self-directed learners, this study provides evidence of the range and nature of the approaches used by students to learn and pass examinations. Although anecdotally educators have been aware that some medical students feel overwhelmed by the vastness of the medical curriculum, and indeed encourage students to form study groups to assist them in preparing for examinations, this study reports for the first time the extent to which an informal, student-driven curriculum exists in one large medical school. Responses by almost 80% of students indicating that they feel overwhelmed by the formal medical curriculum, and yet the perception that the formal curriculum does not provide them with all they need to know to be good doctors should be examined more closely. The study provides evidence of the nature and extent of the learning enterprise that is undertaken by students to meet the demands of the medical program. It has demonstrated that all students access one or more of these informal activities to complement what the medical school program offers. Although the reasons for the use of these informal activities can vary, further research is necessary to establish whether the balance in the formal and informal activities associated with student learning in medical programs occur to further enhance student progress, are completed because of a perceived weakness in the delivery and effectiveness of the formal resources, or are an outcome of the drive to self-directed and life-long learning which are promulgated as outcomes of modern undergraduate medical curricula. The perception that the informal curriculum is needed to the extent suggested in this study, and that it may be supporting strategic and superficial learning approaches, should be a significant point for further discussion amongst curriculum planners to establish why this is the case, and whether there are deficiencies in curricula. Based on the results of this study, it is evident that there needs to be a better alignment between the formally delivered curriculum, and what students perceive is needed to pass the examinations. This alignment is necessary to avoid the potential overload on students with respect to their learning. Curriculum planning teams need to be more responsive to these additional strategies as part of their program review and renewal processes.

## Competing interests

The authors declare that they have no competing interests.

## Authors' contributions

JZ: Made substantial contributions to conception and study design, carried out data collection and performed the statistical analysis and interpretation of data. Has also been involved in drafting the manuscript, revised it critically for important intellectual content and given final approval for the version to be published. RFP: Made substantial contributions to conception and study design. Has also been involved in drafting the manuscript, revised it critically for important intellectual content and given final approval for the version to be published. IZO: Made substantial contributions to study design, data analysis and interpretation of data. Has been involved in drafting the manuscript, revised it critically for important intellectual content and given final approval for the version to be published.

## Pre-publication history

The pre-publication history for this paper can be accessed here:

http://www.biomedcentral.com/1472-6920/11/87/prepub
